# Cell-Free Fat Extract Improves Ovarian Function and Fertility in Mice With Advanced Age

**DOI:** 10.3389/fendo.2022.912648

**Published:** 2022-06-16

**Authors:** Mengyu Liu, Wenzhu Li, Xiaowei Zhou, Mingjuan Zhou, Wenjie Zhang, Qiang Liu, Aijun Zhang, Bufang Xu

**Affiliations:** ^1^ Department of Obstetrics and Gynecology, Ruijin Hospital, Shanghai Jiao Tong University School of Medicine, Shanghai, China; ^2^ Shanghai Key Laboratory of Tissue Engineering, Department of Plastic and Reconstructive Surgery, Shanghai 9th People’s Hospital, Shanghai Jiao Tong University School of Medicine, Shanghai, China; ^3^ Shanghai Key Laboratory of Reproductive Medicine, Department of Histoembryology, Genetics and Developmental Biology, School of Medicine, Shanghai Jiao Tong University, Shanghai, China

**Keywords:** ovarian aging, cell-free fat extract, granulosa cells, cellular senescence, ovarian microenvironment, DNA damage

## Abstract

The reduction in the quantity and quality of oocytes is the major factor affecting fertility in women with advanced age, who tend to experience delayed childbearing and declined fertility rate. However, effective therapeutic strategies to combat this decrease in ovarian function are lacking in clinical practice. Thus, identifying a new method to rescue ovarian function and improve reproduction in natural age-related decline in fertility is necessary. Cell-free fat extract (CEFFE) has been verified to possess diverse active proteins exerting anti-aging and proliferation-promoting effects. Nonetheless, whether CEFFE can rescue the decline in aged-related ovarian function and improve the fertility of females with advanced age remains unclear. In this study, a natural aging mouse model, exhibiting similarities to the physiological changes of ovarian senescence, was used to observe the anti-aging effect of CEFFE on ovarian functions. We found that CEFFE, injected *via* the veins, could recover the levels of the sex hormone, increase angiogenesis and the number of growth follicles in the natural aging mice model. Moreover, CEFFE promoted the development of embryos and increased the litter size of aged mice. Transcriptome analysis of the aged mouse ovaries revealed that CEFFE treatment upregulated the expression of genes involved in the repair of DNA damage. And both *in vivo* and *in vitro* experiment proved that CEFFE improved the function of granulosa cells, including promoting proliferation, alleviating senescence, and rescuing DNA damage in aged granulosa cells. Collectively, our study implied that CEFFE improved the ovarian function and fertility of naturally aging mice by ameliorating the overall microenvironment of ovary, which provided a theoretical basis for new anti-aging therapeutic strategies for cell-free therapy in ovaries.

## Introduction

Globally, approximately 15% of couples (48.5 million) are affected by infertility (1), and the age-related decline in ovarian functions is becoming increasingly common. Generally, ovarian function, principally determined by the number of ovarian follicles and quality of oocytes, begins to experience a decline around 30 years of age (2). In couples not using contraception, the pregnancy rate is 25% per cycle in women aged 20–30 years, while it was less than 5% in females older than 40 years. Meanwhile, the fertility rates decrease, and the rates of aneuploidy and spontaneous abortion increase with increasing age in women (3, 4). However, the increased life expectancy of females worldwide and the tendency of delayed childbearing have rapidly expanded the demand for assisted reproductive technology (5, 6).The percentage of IVF cycles that resulted in live births was 41.5% in women younger than 35 years but only 22.1% in women aged 38–40 years (7).

Ovarian microenvironment, including enriched vasculature, various growth factors, requisite hormone synthesis and normal function of granulosa cells, is vital for the mature of oocytes (8). Maintenance of microenvironment homeostasis is a key factor for normal ovarian function. However, during the aging process, due to the collage deposition and reduced efficiency of tissue remodeling, resulting the imbalance of microenvironment homeostasis and the fibrosis of ovary, eventually lead to the decline of ovarian function (9). Therefore, comprehensive restoration of ovarian microenvironment health is essential to improve ovarian function.

In recent years, many studies have investigated methods to improve ovarian functions, and the effects of stem cells or exosomes on ovarian functions have been well-documented (10). For example, human umbilical cord mesenchymal stem cell-derived exosomes can partly delay ovarian aging in female mice (11). However, immune rejection and ethical risks of stem cell therapy and the limited source, low productivity, and difficulty in scaling up the production of exosomes have limited the clinical application of stem cells and exosomes (12, 13). Several studies have shown that stem cells can contribute to tissue repair *via* secretion of growth factors (14, 15). For example, adipose-derived stem cells can rescue the ovarian function *via* secretion of HGF and bFGF from stem cells (16, 17). Many growth factors were vital for maintaining the balance of ovarian microenvironment and further affecting the functions of ovary, however, the low of cytokines production and expensive treatment costs are not conducive to their wide application (18, 19). Despite the growing attention in ovarian antiaging research, there are no clinically feasible strategies to either preserve or rejuvenate reproductive functions during aging (20, 21).

Adipose tissue, which is rich in cytokines, has been processed and applied in various diseases (22, 23). Cell-free fat extract (CEFFE) is a cell-free liquid with active protein components acquired from adipose tissues *via* the process of mechanical emulsification and centrifugation to remove cellular debris and viable cell components and sterilization, which has advantages of an autogenous source, low immunogenicity, no tumorigenicity, high clinical safety, and low ethical risk. Furthermore, our previous proteomic spectrometry data showed that CEEFE contains more than 1,000 protein components, such as BDNF, HGF, and bFGF (24). Additionally, the anti-oxidant and anti-aging effects of CEFFE on skin repair and promotion of angiogenesis and proliferation on vaginal atrophy have also been reported (25, 26), indicating its potential in anti-aging therapy. However, whether CEFFE can rescue the senescence of ovaries remains unclear. In this study, a natural aging mouse model (10-month-old female mice), which is analogous to 35-40 years of age in human women, was used to observe the anti-aging effect of CEFFE on the ovarian functions of aged mice, as well as the ameliorated effect on ovarian microenvironment. Furthermore, the underlying molecular mechanism of the ovarian anti-aging effect of CEFFE was explored to provide a new therapeutic strategy for rescuing ovarian aging clinically.

## Materials and Methods

### Preparation of CEFFE

CEFFE was provided by SEME Cell Technology Co., Ltd (Shanghai, China), and it was extracted from fresh adipose tissues as previously described (24). Briefly, the fat tissue was washed with saline several times to discard the blood components. Next, the tissue was emulsified mechanically, centrifuged several times to obtain the lowest fluid layer, and stored at –80°C. The protein concentration of CEFFE was measured using a BCA protein kit (Vazyme, China), and the solution was diluted to 3 mg/mL before use.

### Natural Aging Animal Model and Animal Treatment

Specific pathogen free 10-month-old female mice and 8-week-old female and male mice of the C5BL/6J strain were purchased from and housed in Shanghai Branch of Beijing Vital River Laboratory Animal Technologies Co. Ltd., China. All animal experiments were conducted according to the guidelines of Animal Ethics Committee (P2022002). The Control group comprised 8-week-old female mice, and the 10-month-old female mice were equally divided into two groups: the Aged and Aged +CF groups. The mice from the Aged +CF group were treated with 200 μL CEFFE (protein concentration: 3 μg/μL) *via* the tail vein every two days for 2 weeks. At the same time, mice from the Control and Aged groups were injected with the same volume of saline.

### Measurement of Anti-Mullerian hormone (AMH), Estrogen (E_2_), and Follicle Stimulating Hormone (FSH) *via* ELISA

Blood samples from mice were collected and stored at room temperature for 30 min, and they were then centrifuged at 2000 rpm for 10 min. The upper supernatant was harvested to detect the serum concentration of AMH, E_2_, and FSH *via* ELISA (Mlbio, China) as previously described (27).

### Hematoxylin and Eosin Staining of Ovaries and Follicle Counts

Ovaries were harvested and fixed with 4% paraformaldehyde for more than 24 h, and dehydration, wax leaching, and embedding were performed. Ovaries were sliced into 4 μm sections. Subsequently, the sections were dewaxed with ethanol and stained with Hematoxylin and Eosin dye. The maximum longitudinal section of each ovary was selected to count all follicular stages based on the morphological characteristics (28). Finally, the number of primordial, primary, secondary, antral, and atretic follicles were recorded and analyzed.

### Embryo Acquisition and Culture

To evaluate the function of CEFFE on embryonic development in aged mice, mice were treated with ovulation induction after two weeks from the last treatment. Briefly, the mice were intraperitoneally injected with 10 IU of pregnant mare serum gonadotropin (Sansheng, China), and after 48 h, 10 IU of human chorionic gonadotropin (Sansheng, China) was injected. Subsequently, female mice were mated with sexually matured male mice in a 2:1 ratio. After 18 h, female mice were sacrificed, and oocytes were collected from the ampulla. Embryo culture was performed as previously described (29). The numbers of total oocytes, 2-cell embryo rate (number of 2-cell embryos/number of total oocytes retrieved), 4-8 cell embryo rate (4-8 cell embryo number/2-cell oocytes retrieved number), morula rate (morula number/2-cell oocytes retrieved number), and blastocyst rate (blastocyst number/2-cell oocytes retrieved number) were recorded.

### Reproductive Assay

To assess the efficiency of CEFFE involved in the improvement of fertility, reproductive tests were conducted. Two weeks after the last treatment, female mice from different groups were mated with sexually mature male mice in a 2:1 ratio for 2 months, and the fertility information was recorded. Following this, male and female mice were separated for 21 days to confirm the final pregnancy status. During the process, the interval time (the time from mating to birth) and number of offsprings were recorded (30).

### Immunohistochemistry Staining

Ovarian sections from mice were dewaxed, retrieved for antigen, blocked with serum, and incubated in the presence of primary antibodies at 4°C overnight. Subsequently, the slides were incubated with secondary antibodies at room temperature for 1 h. Finally, the target was colored with DAB chromogenic reaction, and the nucleus was counterstained with hematoxylin (Servicebio, China). Images were captured using an optical microscope, and they were analyzed using Image J software. The detail information of antibodies is listed in **Supplementary Table 1**.

### Transmission Electron Microscope (TEM)

The ovaries were collected and fixed in the fixative for TEM (Servicebio, China) in the dark at 4 °C. Following this, the ovaries were dehydrated at room temperature with gradient concentrations of ethanol. Next, the ovaries were penetrated and embedded with resin, and the samples were moved into a preheated 65 °C oven for more than 48 h for polymerization. The resin blocks were cut into 80 nm-thin sections and fished out using 150 meshes. Following this, 2% uranium acetate saturated alcohol solution and 2.6% lead citrate (Servicebio, China) were used to stain the section. Finally, the ovary section was observed under TEM (Hitachi, Japan) and images were captured.

### Immunofluorescence Staining

Cells were fixed with 4% PFA (Servicebio, China) at room temperature for 15 min, permeabilized with 0.1% Triton X-100 for 10 min, and blocked with 5% BSA in PBS for 30 min. Next, the cells were incubated with different primary antibodies at 4 °C overnight. On the second day, the cells were washed with PBST (PBS+0.1% Tween-20) and incubated with Alexa Fluor 488-conjugated rabbit secondary antibody. Subsequently, the nucleus was labeled with Hoechst 33342 for 15 min. Finally, the samples were photographed using a Zeiss-Axio Vert.A1 fluorescence microscope. The detail information of antibodies is listed in **Supplementary Table 1**.

### Total RNA Extraction, Library Preparation, and Sequencing

The ovaries were collected from mice, washed with PBS, immediately frozen in liquid nitrogen, and stored at -80 °C for RNA-seq and RT-PCR. RNA-seq was supported by the Shanghai Silver Crown Biomedical Technology Co., Ltd. Briefly, the total RNA of ovaries was extracted using miRNeasy Mini Kit (QIAGEN, USA), and the concentration and integrity of RNA were analyzed *via* NanoDrop and Agilent 4200 TapeStation (Agilent Technologies, USA), respectively. Only the RNAs with RNA integrity values between 8 and 10 and purity between 1.8 and 2.1 were included in this study. The RNA-Seq library preparation and sequencing were performed using Illumina NovaSeq 6000 (Illumina, USA). Three biological replicates were used for RNA-Seq. The cutoffs of Fold Change ≥ 1.5(|log2 ratio| ≥ 0.58) and P adjust < 0.05 were applied to identify the differentially expressed genes (DEGs) between every two groups. The DEGs were further analyzed *via* Venn analysis and Gene Ontology (GO) & Kyoto Encyclopedia of Genes and Genomes (KEGG) enrichment analyses.

### RNA Extraction and qRT-PCR

Total RNA was extracted from different ovaries or cells using Total RNA Isolation kit (Vazyme, China). The conversion of RNA to complementary DNA was performed using Hiscript II Reverse Transcriptase (Vazyme, China) according to manufacturer’s protocol, and the relative quantitative analysis was performed *via* QuantStudio TM 6 Flex (Applied Biosystems, USA). The synthesized cDNA was amplified and detected using ChamQ SYBR qPCR Master Mix (Vazyme, China). A comparative threshold cycle was normalized using GAPDH, and it was analyzed using the 2^-ΔΔCT^ method to compare the mRNA expression of different samples. The primer sequences are listed in **Supplementary Table 2**.

### Primary Human Granulosa Cell Collection and Cell Culture

The procedure of primary human granulosa cell collection was approved by the Institutional Review Board of Reproductive Medicine of Ruijin Hospital. The follicular fluids were collected during oocyte retrieval from women with the age less than 35 who sought infertility treatment due to tubal obstruction or male factors with informed consent, approved by the ethics committee of Ruijin Hospital (2020104a). And our study conformed the Enhancing the QUAlity and Transparency Of health Research (EQUATOR) network guidelines. The patients with abnormal chromosome, endometriosis, polycystic ovarian syndrome and other diseases which could affect folliculogenesis were excluded. The granulosa cells were collected from follicular fluids using density gradient centrifugation with lymphocyte separation medium (Solarbio, China). Firstly, the follicular fluid was centrifuged to collect the sediment, and it was then washed with PBS once. The cells were resuspended with PBS, transferred to the lymphocyte separation medium in an equal volume ratio, and then centrifuged at 1000 g for 20 min. Next, the medium layer cells were carefully collected and washed with PBS. Subsequently, red blood cells were discarded with Red Cell Lysis Buffer (Solarbio, China), and they were digested with 1 mg/mL hyaluronidase (Yeasen, China). Finally, the cells were resuspended in complete medium, and they were cultured in a dish precoated with 0.2% gelatin at 37 °C for 1 h. KGN cell line was purchased from Feiya Biotechnology Co., Ltd., China. Both human primary ovarian granulosa cells (hGCs) and KGN cells were cultured in DMEM/F12 medium containing 10% FBS (Gibco, USA), 100 U/mL penicillin, and 100 µg/mL of streptomycin (Solarbio, China). Besides, during the culture of hGCs, recombinant human follitropin (Switzerland) was added, and both cells were maintained under 5% CO_2_ at 37°C. To induce DNA damage models of KGN and hGCs *in vitro*. 10 μM camptothecin (CPT, MedChemExpress, U.S) was added to the cell culture medium and incubate for 2 h. Then the cell culture medium was changed and cells were further treated with/without CEFFE.

### Senescence-Associated β-galactosidase Staining

The cells were fixed with fixed liquid at room temperature for 15 min. After washing with PBS thrice, the cells were incubated with β-galactosidase staining solution at 37 °C without CO_2_ overnight. On the second day, cells were observed and pictured under optical microscope, and the senescence cells showed blue-green color.

### Proliferation Assay With CCK-8 and EdU Test

The CCK-8 assay kit (Yeasen, China) was used to observe cell growth. KGN cells were plated in 96-well plates at 24 h before treatment. At the end of the treatment period, 10 µL of CCK-8 reagent was added to each well, and it was incubated in a 5% CO_2_ atmosphere at 37°C for 1 h. The optical density was then measured using a Multiskan GO instrument at 450 nm.

To analyze the effect of CEFFE on the proliferation of human primary granulosa cells, EdU assay was performed according to manufacturer’s instructions using fluorescent microscope. Green fluorescent labeled cells represented the cells that possessed proliferative activity and blue fluorescent labeled the nucleus of all cells. The number of cells labeled with different fluorescence markers was counted using Image J, and the ratio of Green/Blue was calculated.

### Western Blotting

Proteins were extracted from cells using RIPA cell lysis, and they were degenerated with 5× protein loading. The proteins were separated and transferred using sodium dodecyl sulfate polyacrylamide gel electrophoresis (Epizyme, China) and 0.2-μm polyvinylidene difluoride membranes (Millipore, USA). Subsequently, the membrane with proteins was incubated with primary and secondary antibodies as described in **Supplementary Table 1**.

### Statistical Analysis

Every experiment was repeated at least three times. All experimental data were expressed as mean ± SD, and it was analyzed using GraphPad Prism 8.0. Two groups were compared using Student’s t-test, and the difference of more than two groups was analyzed *via* one-way analysis or nonparametric Kruskal-Wallis test according to the variances. *P* value of less than 0.05 was considered statistically significant.

## Results

### CEFFE Improves the Ovarian Function of Naturally Aged Mice

**Figure 1:**
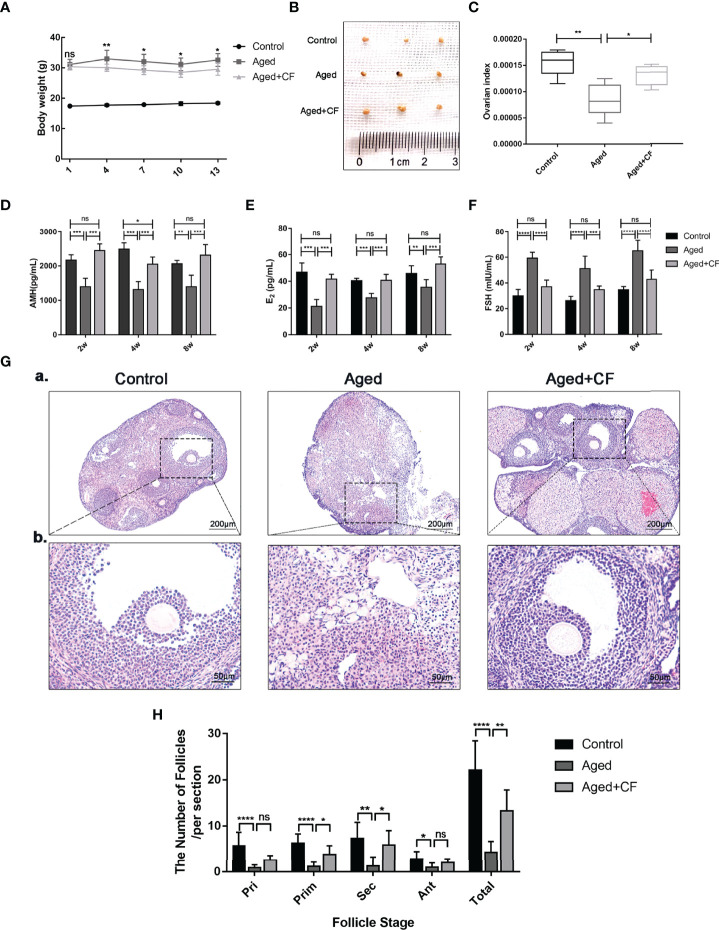
Cell-free fat extract (CEFFE) improves ovarian function in aged mice. **(A)** The body weight of mice was monitored every two days during the treatment duration (n=5). **(B)** Gross morphologies of dissected ovaries from different groups (n=3). **(C)** The index of ovaries (the ratio of ovarian weight to body weight) in each group were compared and analyzed (n=5). **(D–F)** Serum AMH, E_2_, and FSH secretion levels were measured *via* ELISA every two weeks until 8 weeks after treatment (n=3-5). **(G)** Representative ovary morphology (a) and follicle morphology (b) of Hematoxylin and Eosin staining from each group were captured. Scale bar=200 μm or 50 μm. **(H)** The number of different stage follicles was calculated and analyzed (n=7). Pri, primordial follicles; Prim, primary follicles; Sec, secondary follicles; Ant, Antral follicles; Atr, atretic follicles; Total, The total number of health follicles (including primordial, primary, secondary and antral follicles). Data are represented as mean ± SD, ns: *P* > 0.05, ^*^ *P* < 0.05, ^**^ *P* < 0.01, ^***^ *P* < 0.001, and ^****^ *P* < 0.0001.

Aged mice are characterized by obesity, ovarian atrophy, and decreased ovarian function. We monitored the change in body mass of mice during CEFFE treatment for two weeks, and the results showed that the body mass in the Aged+CF group gradually decreased compared to that in the Aged group (**Figure 1A**). Meanwhile, the diet, mental state, and activity of Aged+CF mice were not affected compared to those of Aged and Control mice. Besides, after 2 weeks of treatment, the weight of ovaries, sex hormones, and follicle count in ovaries were analyzed. Higher ovary weight (Aged+CF vs Aged: 95% CI, 0.02 to 2.28 mg; P<0.05) (**Figures 1B, C**), increased AMH and E_2_ levels and decreased FSH levels were observed in the Aged+CF group than in the Aged group (**Table 1**). Besides, the recovery effect of CEFFE on sex hormones lasted up to 2 months after treatment (**Figures 1D–F**). All these indices showed an opposite trend in the Aged group than that in the Control group. The above results suggested that CEFFE could promote the recovery of hormone synthesis function in aged mice, which is responsible for regulating the stability of ovarian microenvironment and stimulating the mature of oocytes. Furthermore, ovaries were stained with Hematoxylin and Eosin to assess the changes in ovarian morphology and follicular number. As expected, in the Aged group, the ovaries exhibited a significantly reduced number of follicles at all stages. After treatment with CEFFE for 2 weeks, many more growth follicles appeared in the Aged+CF group than in the Aged group, especially the primary (95% CI, 0.44 to 4.91; P<0.05) and secondary follicles (95% CI, 1.02 to 8.19; P<0.05) (**Figures 1G, H**). Besides, granulosa cells were more regularly arranged in Aged+CF group as compared to that in Aged group. In conclusion, CEFFE could improve the function of aged ovaries on the size of ovaries, hormone secretion, and number of follicles.

**Table 1:** The *P* value and 95% CI of diff in AMH, E_2_ and FSH analysis.

	AMH (pg/mL)			
	Control vs Aged		Aged+CF vs Aged	
	*P* value	95% CI of diff (pg/mL)	*P* value	95% CI of diff (pg/mL)
2 w	<0.001	387.69 to 1156.80	<0.001	688.69 to 1413.82
4 w	<0.001	812.74 to 1537.87	<0.001	375.07 to 1100.20
8 w	0.001	248.46 to 1086.77	<0.001	531.77 to 1300.89
	E_2_ (pg/mL)			
	Control vs Aged		Aged+CF vs Aged	
	*P* value	95% CI of diff (pg/mL)	*P* value	95% CI of diff (pg/mL)
2 w	<0.001	18.02 to 32.97	<0.001	12.92 to 27.87
4 w	<0.001	5.30 to 20.25	<0.001	5.61 to 20.56
8 w	0.008	2.45 to 18.31	<0.001	9.46 to 25.31
	FSH (mIU/mL)			
	Control vs Aged		Aged+CF vs Aged	
	*P* value	95% CI of diff (mIU/mL)	*P* value	95% CI of diff (mIU/mL)
2 w	<0.0001	-38.51 to -20.14	<0.0001	-31.45 to -13.08
4 w	<0.0001	-34.16 to -15.79	0.0003	-25.63 to -7.26
8 w	<0.0001	-40.02 to -20.53	<0.0001	-31.98 to -12.50

CI, Confidence Interval; AMH, Anti-Mullerian hormone; E_2_, Estrogen; FSH, Follicle Stimulating Hormone.

### CEFFE Improves the Quality of Oocytes Derived From Aged Mice and Fertility

**Figure 2:**
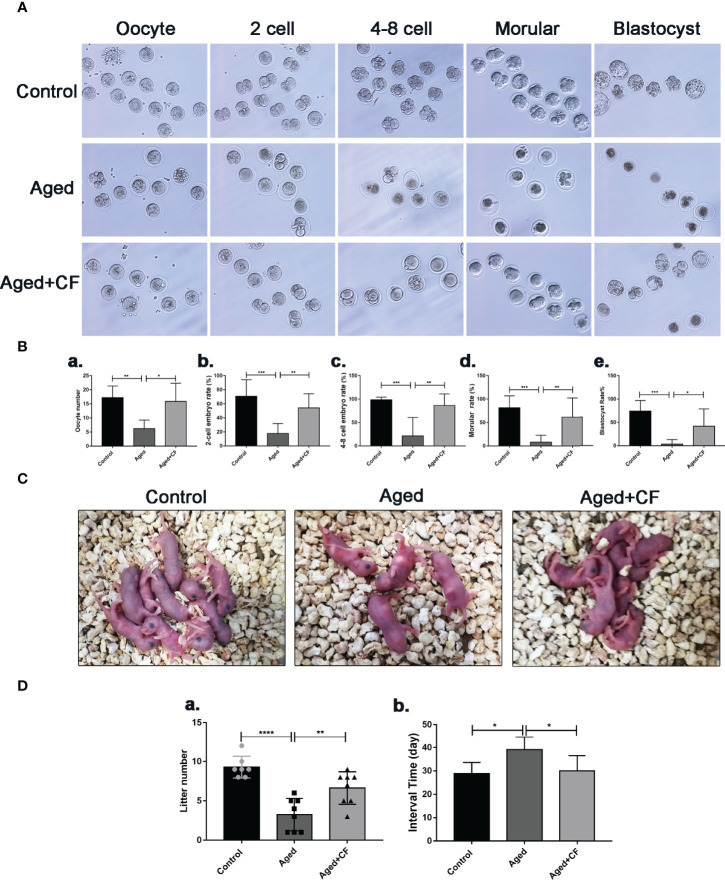
The impact of CEFFE treatment on fertility restoration. **(A)** Representative morphology of embryo development in different groups, including the oocyte, 2 cell embryo, 4-8 cell embryo, morula, and blastocyst stages. **(B)** Total number of oocytes and the rate of 2 cell embryo, 4-8 cell embryo, morula, and blastocyst were recorded in different groups (n=6-7). **(C)** Representative images of litters from Control, Aged, and Aged+CF groups (n=7-8). **(D)** Statistical analysis of litter numbers (a) and the interval time (the time from mating to birth) (b) Data are represented as the mean ± SD, ^*^ *P* < 0.05, ^**^ *P* < 0.01, ^***^ *P* < 0.001, and ^****^ *P* < 0.0001.

As indicated by the above findings, CEFFE could improve the ovarian function. To further investigate the effect of CEFFE on the fertility of mice, we collected oocytes from the ampulla of female mice after 18 h of intercourse, and we cultured them *in vitro* to observe and record the process of embryo development. The results showed that more oocytes, 2 cell embryos, 4-8 cell embryos, morulas, and blastocysts were observed in the Aged+CF group than in the Aged group, indicating that CEFFE could enhance the development of embryos (**Figures 2A, B**). Next, we performed natural fertility assays in each group to evaluate the litter size. The litter size was the highest and the time from mating to birth was the shortest in the Control group. Compared to the Aged group, mice from Aged+CF group had a greater litter size and shorter time from mating to birth (**Figures 2C, D**). The detail results were displayed in **Table 2**. So far, we found that CEFFE significantly improved the number of retrieved oocytes, embryonic developmental ability, and final litter size, indicating that CEFFE significantly improved the fertility of aged mice.

**Table 2:** The statistics of oocyte number, 2 cell embryo rate, 4-8 cell embryo rate, morula rate, blastocyst rate, litter number and interval time.

	Control	Aged	Aged+CF	Aged vs Control		Aged+CF vs Aged	
				*P* value	95% CI of diff	*P* value	95% CI of diff
Oocyte number	17.22 ± 4.12	6.20 ± 3.03	15.83 ± 6.49	< 0.0001	-23.94 to -11.85	0.0033	2.99 to 15.08
2 cell embryo rate (%)	70.71 ± 23.57	17.91 ± 13.95	54.24 ± 20.08	0.0004	-80.41 to -25.18	0.0071	9.80 to 62.86
4-8 cell embryo rate (%)	98.53 ± 5.60	21.43 ± 39.34	86.31 ± 24.85	0.0003	-116.8 to -37.41	0.0012	26.75 to 103.02
Morula rate (%)	81.59 ± 25.37	8.33 ± 14.43	61.31 ± 40.95	0.0009	-114.98 to -31.53	0.0093	12.89 to 93.07
Blastocyst rate (%)	74.35 ± 22.65	3.57 ± 9.45	41.67 ± 37.27	0.0002	-105.49 to -36.06	0.0405	1.56 to 74.63
Litter number	9.28 ± 1.38	3.25 ± 2.05	6.63 ± 2.07	< 0.0001	-8.50 to-3.57	0.0050	0.99 to 5.76
Interval time(days)	28.83 ± 4.75	39.14 ± 5.40	30.00 ± 6.57	0.0118	2.26 to 18.36	0.0252	-17.2 to -1.09

CI, Confidence Interval.

### CEFFE Contributes to the Stability of Ovarian Microenvironment in Aged Ovaries

**Figure 3:**
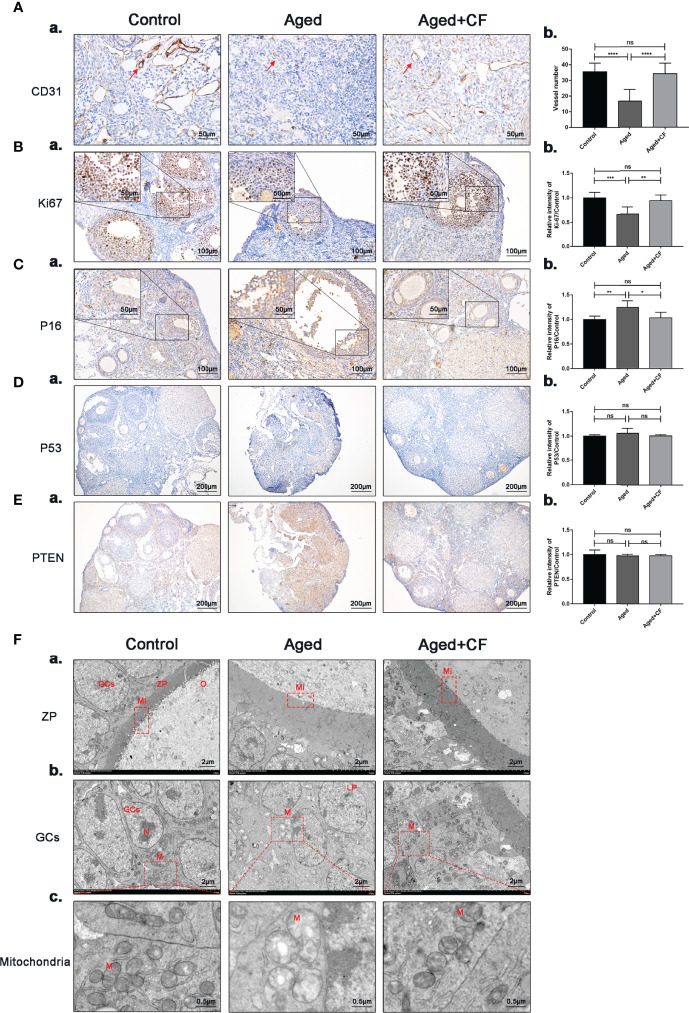
CEFFE promotes angiogenesis in the medulla, promotes proliferation, and reduces senescence in ovarian granulosa cells. **(A)** IHC staining of the angiogenesis marker CD31 of ovarian sections (a) and statistical analysis (b) (n=4), the red arrows indicate blood vessels. Scale bar=50 μm. **(B)** IHC staining of the proliferation marker Ki67 of ovarian sections (a) and statistical analysis (b) (n=5-7), scale bar=100 μm. There are partial enlarged images of granulosa cells in the upper left corner of figure (a), scale bar=50 μm. **(C)** IHC staining of the senescence marker P16 of ovarian sections (a) and statistical analysis (b) (n=4), scale bar=100 μm. There are partial enlarged images of granulosa cells in the upper left corner of figure (a), scale bar=50 μm. **(D)** IHC staining of the suppressive gene PTEN of ovarian sections (a) and statistical analysis (b) (n=4), scale bar=200 μm. **(E)** IHC staining of the oncogenic gene P53 of ovarian sections (a) and statistical analysis (b) (n=4), scale bar=200 μm. **(F)** Effects of CEFFE treatment on the ultrastructural changes of zona pellucida (a), granulosa cells (b), and mitochondria (c) were observed *via* TEM. Scale bar=2 μm or 0.5 μm. O, Oocyte; GCs, granulosa cells; ZP, Zona pellucida; Mi, microvilli; LD, Liquid droplet; N, Nucleus; M, mitochondria. Data are represented as the mean ± SD, ns: *P* > 0.05, ^*^ *P* < 0.05, ^**^ *P* < 0.01, ^***^ *P* < 0.001, and ^****^ *P* < 0.0001.

The ovary was mainly composed of the medulla, rich in blood and lymphatic vessels, and cortex, rich in different stage follicles. In aged ovaries, the number of microvessels in the medulla decreased due to medullary fibrosis. Vessels could provide sufficient nutrients for follicle development, and they were essential for the maintenance of ovarian microenvironment. Therefore, expression of the vascular endothelial marker CD31 was detected *via* IHC, and the results showed that angiogenesis significantly decreased in the Aged group (Aged vs Control: 95% CI, -26.68 to -11.10; P<0.0001) whereas it significantly increased after treatment (Aged+CF vs Aged: 95% CI, 9.55 to 26.51; P<0.0001), suggesting that CEFFE could promote ovarian medulla angiogenesis in aged mice (**Figure 3A**). Next, proliferation and senescence were evaluated with Ki67 and P16 *via* IHC analysis. In the Aged group, expression of the proliferation marker Ki67 decreased (Aged vs Control: 95% CI, -0.51 to -0.16; P<0.001) and that of the senescence marker P16 increased in GCs (Aged vs Control: 95% CI, 0.07 to 0.41; P<0.01). After treatment with CEFFE, more proliferative (Aged+CF vs Aged: 95% CI, 0.11 to 0.43; P<0.01) and less senescent GCs (Aged+CF vs Aged: 95% CI, -0.42 to -0.01; P<0.05) were observed (**Figures 3B, C**). Additionally, to rule out the tumorigenic risk of CEFFE treatment, the expression of tumor marker P53 (oncogenic gene) and PTEN (suppressive gene) was analyzed, and the results displayed no difference between each group, indicating that CEFFE had no tumorigenic risk for ovarian function recovery (**Figures 3D, E**). It is well known that during aging, the morphology and number of mitochondria change, which are vital for maintaining cell function. To observe changes in the internal microstructure of cells, ovary TEM was performed. In zona pellucida, the microvilli between inner zona pellucida and oocytes, which are vital for material and message exchange between granulosa cells and oocytes in aged ovary, were seriously degenerated. However, the bidirectional granulosa cells-oocytes signaling is critical for creating a dynamic intrafollicular microenvironment (8).In granulosa cells, more vacuoles and swollen mitochondria with less cristae were observed in the aged ovary. Interestingly, in the Aged+CF group, the microvilli were integrated, and mitochondrial morphology was clear with more cristae. These results strongly indicated that CEFFE treatment effectively ameliorated the microstructure disorder of ovary (**Figure 3F**). Above all, we demonstrated that CEFFE could promote angiogenesis in medulla and alleviate the senescence of granulosa cells.

### Mouse Ovarian Transcriptome Sequencing *via* RNA-Seq

**Figure 4:**
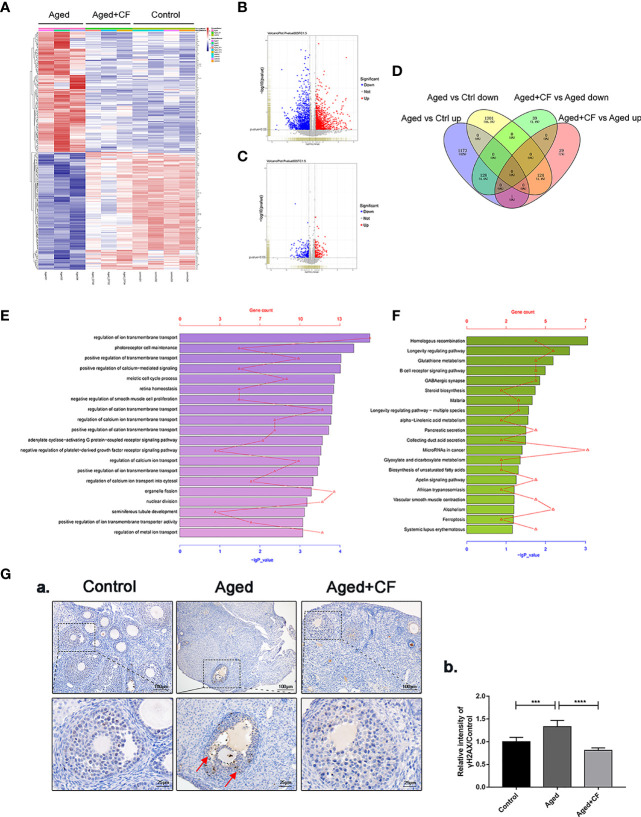
Transcriptome and Bioinformatics Analyses of Ovaries. **(A)** Heatmap of differentially expressed genes (DEGs) from Aged vs Aged+CF vs Control group with absolute fold change of more than 1.5 and *P* value of less than 0.05. Aged group: n=3, Aged+CF group: n=3, Control group: n=4 **(B)** The volcano plot of DEGs of Aged vs Control. Among the total 2,727 genes, 1,302 genes were upregulated while 1,425 genes were downregulated. **(C)** The volcano plot DEGs of Aged+CF vs Aged. Among the total 321 genes, 154 genes were upregulated while 167 genes were downregulated. **(D)** Venn analysis of Aged vs Control and Aged+CF vs Aged group, following which, 252 DEGs with opposite trends in the two clusters were selected. **(E)** DEGs derived from Venn analysis were further analyzed *via* GO analysis. **(F)** DEGs derived from Venn analysis were further analyzed *via* KEGG analysis. **(G)** IHC staining of γH2AX of ovarian sections (a) and statistical analysis (b) (n=5), scale bar=100 μm or 25 μm. Data are represented as the mean ± SD, ^***^ *P* < 0.001, and ^****^ *P* < 0.0001.

We performed transcriptome sequencing and differential gene expression analysis on ovaries from the Control, Aged, and Aged+CF groups to find the main signal pathways with changes. Firstly, by comparing the transcriptome data of each of the two groups (Aged vs Ctrl and Aged+CF vs Aged, respectively), only the genes with absolute fold change >1.5 and *P* value < 0.05 were defined as DEGs (**Figures 4A–C**). Next, the intersection of the two clusters was performed *via* Venn analysis, and a total of 252 genes that showed opposite trends were selected in the two clusters (**Figure 4D**). The GO pathway analysis showed that the 252 DEGs were most enriched in “transmembrane transport” of the top 20 pathways, which might be related to the process of active proteins in CEFFE interacting with cells (**Figure 4E**). The KEGG pathway analysis showed that the 252 DEGs were enriched in pathways related to cell senescence, including “Homologous recombination”, “Longevity regulating pathway”, and “Glutathione metabolism” (**Figure 4F**). Among these, bioinformatic analysis strongly suggested that homologous recombination (HR), an essential method for DNA double-strand break (DSB) repair, was significantly enriched, indicating that CEFFE could repair the ovarian function in aged mice by promoting DSB repair. To further confirm the change in DNA damage repair *in vivo*, γH2AX was detected in the ovaries *via* IHC, and more γH2AX-positive cells were observed in the Aged group than in the Control and Aged+CF groups (Aged vs Control: 95% CI, 0.16 to 0.50; P<0.001), (Aged+CF vs Aged: 95% CI, -0.69 to -0.35; P<0.0001), especially in granulosa cells (the red arrows indicate γH2AX positive cells) (**Figure 4G**). Collectively, the RNA-Seq and bioinformatic analyses indicated that DNA damage was most obviously changed in the ovaries with CEFFE treatment, which might play an essential role in rescuing the ovarian function.

### CEFFE Promotes the Proliferation of Senescent KGN Cells Induced by D-gal *In Vitro*

**Figure 5:**
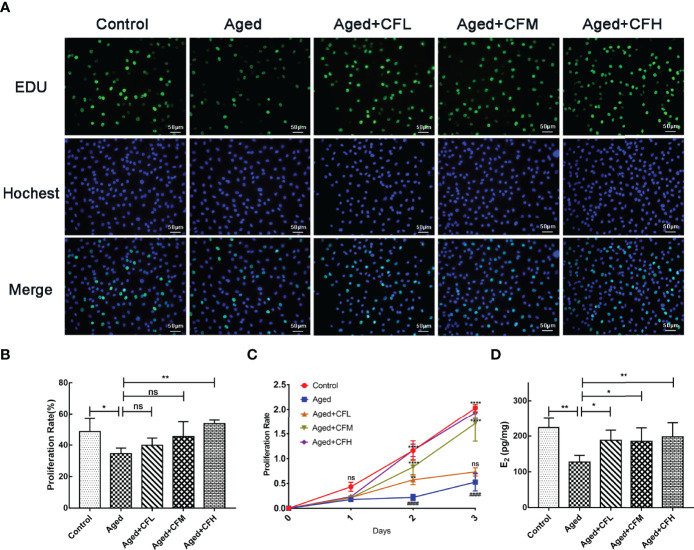
CEFFE recovers proliferation and E_2_ secretion of senescent KGN cells. **(A)** Proliferation of KGN cells was measured *via* EdU assay. Proliferative cells were labeled *via* green fluorescence, and all nuclei were labeled *via* blue fluorescence. Scale bar=50 μm. **(B)** Statistics of proliferative cell rates. **(C)** Proliferation of KGN cells was measured using the CCK-8 assay at 24 h, 48 h, and 72 h. **(D)** The E_2_ content in cellular supernatant was measured *via* ELISA, and the results were standardized with the concentration of KGN cell proteins in each group. Three independent experiments were conducted in each group, and data are represented as the mean ± SD, ns: *P* > 0.05, ^*^ *P* < 0.05, ^**^ *P* < 0.01, ^****^ *P* < 0.0001, ^####^P < 0.0001 ^*^Compared with Aged group, ^#^Compared with Control group.

Next, we established an *in vitro* model of senescent human granulosa cell line (KGN) through D-gal treatment. Senescence β-Galactosidase Staining Kit was used to examine the expression of β-Galactosidase, which increased during senescence, and the results showed that 10 mg/mL and 20 mg/mL D-gal intervention for seven days increased the number of SA-β-gal-positive cells (**Supplementary Figure 1A**). Additionally, the expression of senescence markers P16 and P21 significantly increased following D-gal treatment compared to that in the Control group (**Supplementary Figure 1B**). Finally, treatment with 10 mg/mL D-gal for 7 days was used to establish the senescent KGN model. Following this, senescent KGN was incubated with three different concentrations of CEFFE (CFL: 0.06 μg/μL, CFM: 0.15 μg/μL, CFH: 0.3 μg/μL) for 24 h, and EdU test was performed. The results showed that the Aged group was less proliferative than the Control group (95% CI, -26.37 to -1.59%; P<0.05), and only CFH could increase the proliferation of aged KGN cells at 24 h (95% CI, 6.03 to 32.15%; P<0.01), (**Figures 5A, B**). To further analyze whether CEFFE could affect the aged KGN proliferation, we prolonged the co-culture time to 72 h. The results showed that CEFFE could promote the proliferation of senescent KGN in a dose- and time-dependent manner (**Figure 5C**). Further, E_2_ content in cellular supernatant was measured *via* ELISA test with CEFFE intervention for 48 h, and the data demonstrated that senescent KGN secreted less E_2_ (Aged vs Control: 95% CI, -160.21 to -32.89 pg/mg; P<0.01) and CEFFE could significantly promote E_2_ secretion (95% CI: Aged+CFL vs Aged, 5.80 to 116.07 pg/mg, P<0.05; Aged+CFM vs Aged, 0.31 to 117.26 pg/mg, P<0.05; Aged+CFH vs Aged, 16.33 to 126.60 pg/mg, P<0.01), (**Figure 5D**). Taken together, our findings suggested that CEFFE contributed to the proliferation and hormone secretion of senescent KGN cells in a dose-dependent manner.

### CEFFE Can Ameliorate KGN Cells and hGCs DNA Damage and Senescence *In Vitro*

**Figure 6:**
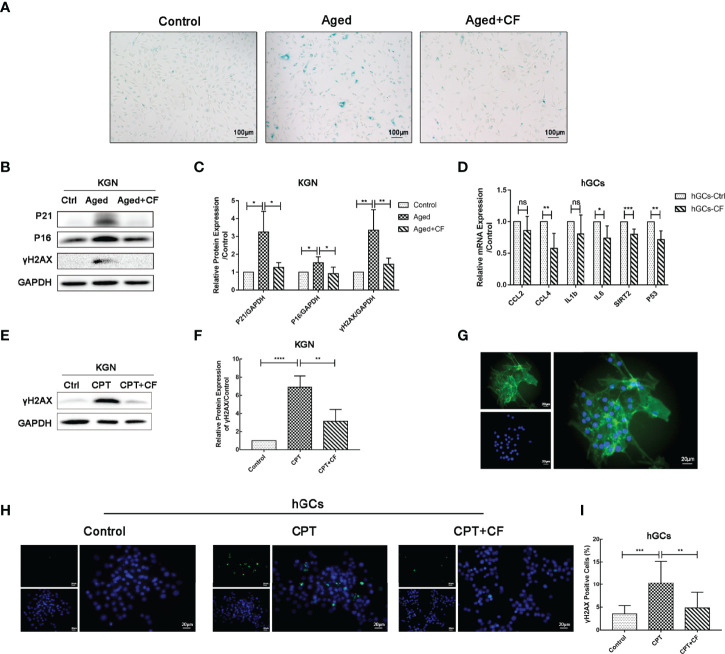
CEFFE resists DNA damage and alleviates the senescence of KGN cells and hGCs. **(A)** Representative images of SA-β-gal staining. Scale bar=100 μm. **(B)** The senescent markers P21 and P16 and DNA damage marker γH2AX were detected *via* Western Blotting. **(C)** The protein expression of P16, P21, and γH2AX was analyzed. **(D)** qRT-PCR analysis of age-related gene expression in hGCs. **(E)** After being exposed to CPT and following treatment with/without CEFFE, γH2AX was detected *via* Western Blotting in KGN cells. **(F)** The protein expression of γH2AX was analyzed in KGN cells. **(G)** hGCs were identified using FSHR protein, which was specifically located in the cytoplasm of hGCs. FSHR was labeled with green fluorescence, and the nucleus was labeled with blue fluorescence. Scale bar=20 μm. **(H)** The DNA damaged marker γH2AX was detected in hGCs induced by CPT with the immunofluorescence method. γH2AX was labeled with green fluorescence, and the nucleus was labeled with blue fluorescence. Scale bar=20 μm. **(I)** Statistics of γH2AX-positive hGCs. CPT, camptothecin. Three independent experiments were conducted in each group, and data are represented as the mean ± SD, ns: *P* > 0.05, ^*^ *P* < 0.05, ^**^ *P* < 0.01, ^***^ *P* < 0.001, and ^****^ *P* < 0.0001.

To examine the effect of CEFFE on KGN senescence, CEFFE was incubated for 48 h, and it was used in the follow-up experiment. As shown in **Figure 6A**, CEFFE could significantly reduce the number of SA-β-gal-positive cells compared to those in the Aged group. Meanwhile, the results of Western Blotting also demonstrated that the expression levels of senescent markers P21 (Aged vs Control:95% CI, 0.59 to 3.91; P<0.05) and P16 (Aged vs Control: 95% CI, 0.12 to 0.93; P<0.05) and DNA damaged marker γH2AX (Aged vs Control: 95% CI, 1.05 to 3.67; P<0.01) increased in the Aged group while they decreased in the Aged+CF group (Aged+CF vs Aged: P21, 95% CI, -3.65 to -0.33, P<0.05; P16, 95% CI, -1.02 to -0.21, P<0.01; γH2AX, 95% CI, -3.22 to -0.60, P<0.01), (**Figures 6B, C**). To further verify the anti-senescent effect of CEFFE, human primary ovarian granulosa cells (hGCs) were cultured with/without CEFFE *in vitro*, and the mRNA expression of some senescence-related genes was detected *via* RT-PCR. Interestingly, CEFFE could significantly reduce the expression of senescence-related genes in hGCs, which was consistent with KGN cells (**Figure 6D**). Next, given the essential role of CEFFE in HR pathway upon DSBs indicated by the transcriptome data in aged ovary, we examined the effect of CEFFE on the repair of camptothecin-induced DNA damage, which could only be rescued by HR but not by non-homologous end joining pathway, another method for DSBs. Briefly, KGN was incubated with 10 μM camptothecin for 2 h to induce DSBs (31). Subsequently, CEFFE was added and co-cultured for 2 h. γH2AX, a sensitive marker of DSBs, was measured *via* Western Blotting, and it was found to be significantly decreased with CEFFE intervention (CPT+CF vs CPT: 95% CI, -5.72 to -1.78; P<0.01), (**Figures 6E, F**). Meanwhile, the hGCs were identified with FSHR (**Figure 6G**) and similar results were observed in hGCs (CPT+CF vs CPT: 95% CI, -9.23 to -1.80%; P<0.01), (**Figures 6H, I** **).** Taken together, our results indicated that CEFFE could promote the repair of DNA damage and reverse cellular senescence of KGN cells.

## Discussion

Currently, subfertility caused by decreased ovarian function is becoming an intractable clinical issue, increasing the difficulty of assisted reproductive technology treatment. Although numerous studies on stem cells, exosomes, and antioxidants have been conducted to improve ovarian function (32, 33), there is still a lack of effective clinical programs to save ovarian function and fertility. In this study, for the first time, we found that CEFFE rescued ovarian function and improved fertility in aged mice by repairing ovarian microenvironment, including angiogenesis, hormone synthesis and the function of granulosa cell in ovaries, which might provide a novel strategy for ovarian anti-aging treatment.

The female reproductive system is of great significance to the women’s health. Declined ovarian function seriously reduces the reproductive lifespan, increases the risk of diseases, and even affects female lifespan (34). Aging of the female reproductive system occurs approximately 10 years prior to the natural age-associated functional decline of other organ systems, and natural fertility ceases approximately 10 years before the onset of menopause (35). After 30 years of age, female fertility declines rapidly, and almost half the women are infertile by the age of 40 years (7, 36). Nowadays, an increasing number of women are delaying childbearing an older age, resulting in an increased demand for assisted conception techniques. This is particularly important to improve the ovarian function in 35–40 years old females. Therefore, 10-month-old mice, were selected as the research model. In this study, CEFFE improved the ovarian size and sex hormone levels in aged mice. Subsequent assessment of fertility demonstrated a slight increase in the number of follicles in the ovarian sections (**Figure 1**). It is well-known that the quantity and quality of oocytes significantly decline with age. To evaluate the quality of oocytes from aged mice, embryonic culture was conducted, and the data indicated that CEFFE increased the number of retrieved oocytes and blastocyst formation rate. Further, mating tests revealed that CEFFE increased the litter size of aged mice. So far, we comprehensively demonstrated that CEFFE could raise the fecundity of aged mice from the number of retrieved follicles and oocytes, embryonic development, and even the litter size (**Figure 2**). With delayed childbearing in women, pregnancy complications and the risk of congenital defects in offspring pose a considerable risk to the health of both mother and child (37). Therefore, to further exclude the tumorigenicity and side effects of CEFFE, biological safety was proven by evaluating tumor markers in the ovary (**Figure 3**). The above results fully proved the effectiveness and safety of CEFFE in ovarian anti-aging therapy and improving fecundity.

Aging is a major determinant of reproductive wellbeing. This communication between the microenvironment, somatic cells, and germ cells is critical for maintaining an appropriate reproductive lifespan in mammals. In this study, we comprehensively evaluated the repair effect of CEFFE on ovarian microenvironment from hormone synthesis, angiogenesis, cell senescence, proliferation, and mitochondrial morphology. In aged ovary, reduced angiogenesis of medulla, increased senescence, weakened proliferation, and abnormal mitochondrial morphology of granulosa cell were observed, and these aging-related phenotypes were significantly ameliorated following CEFFE treatment. To further reveal the underlying anti-ovarian aging mechanism of CEFFE, ovaries from Control, Aged, and Aged+CF groups were analyzed *via* mRNA transcriptome sequencing. Following this, Venn and KEGG analyses were performed, suggesting that DNA HR, an important method for DNA damage repair (DDR), was significantly enriched (**Figure 4**). It has been recognized that increased DNA damage and repair deficiency in granulosa cells facilitate follicle atresia and ovarian aging (38). Similarly, in our study we observed a higher amount of DNA damaged GCs in aged ovaries, and this damage significantly reduced after CEFFE treatment *via* IHC analysis, suggesting that CEFFE could repair the DNA damage of granulosa cells (**Figure 4**).

Ovarian granulosa cell aging is significantly associated with ovarian dysfunction and impaired fertility (34, 35). Meanwhile, DNA damage significantly affects the normal physiological function of cells, which is a key pathway regulating reproductive aging in the genome-wide association study (39), single-gene study of premature ovarian failure (40), and animal model study (41). Moreover, the damaged granulosa cells were adverse to maintain the homeostasis of ovarian microenvironment. To explore the effect of CEFFE on the function of granulosa cells, we constructed a galactose-induced senescent granulosa cell model *in vitro* for further validation. The results suggested that CEFFE could promote the proliferation and repair of DNA damage in aged KGN cells. What’s more, CEFFE also alleviated the senescence and promoted the repair of DNA damage of human primary ovarian granulosa cells. (**Figures 5**, **6**). Importantly, for the first time, we found that CEFFE could repair the function of aged granulosa cells. Our previous studies have shown that CEFFE is rich in various growth factors, such as PDGF, BDNF, GDNF, and TGFβ, which are also abundant in the follicular fluid, contributing to the regulation of intrafollicular microenvironment and the maturation of granulosa cells and follicles (15). In contrast, PDGF and GDNF contents in the follicular fluid of patients with ovarian dysfunction (such as DOR and aging) were lower than those in young people with normal ovarian functions (42). Besides, BDNF (43), TGFβ (44), IGF (45), and other cytokines have also been reported to be involved in DDR. Combined with the above results, we proposed that CEFFE could reduce the senescence and recovery the function of granulosa cells probably by repairing DNA damage in the ovary.

Our study found that CEFFE improved the ovarian function and fertility of mice with advanced age for the first time, probably by improving the function of granulosa cells and ameliorating the overall microenvironment of ovary. Besides, our study also provided a novel strategy for rescuing the ovarian function that diminished with age, which could reduce the economic and emotional burden of infertility treatment and increase the success rate of assisted reproductive technology treatment in women with advanced age (46). However, there also some limitations in our study, the specific molecules in CEFFE, which played an essential role in the ovarian function repair still need further investigation, which is conducive to finding effective molecular combinations and providing more efficient and precise treatment for ovary aging.

## Conclusion

For the first time, we investigated the anti-aging effect of CEFFE on the aged ovaries and found that CEFFE effectively improved the ovarian function and fertility of aged mice by ameliorating the overall microenvironment of ovary, which provided a theoretical basis for novel anti-aging strategies of cell-free therapy in ovaries and even other age-related diseases. However, the specific molecules and precise repair mechanism in CEFFE still need further investigation, meanwhile, related clinical research also need to be proceeded to overall assessment the effect of CEFFE on ovary function in the future.

## Data Availability Statement

The data discussed in this publication have been deposited in NCBI’s Gene Expression Omnibus and are accessible through GEO Series accession number GSE202124 (https://www.ncbi.nlm.nih.gov/geo/query/acc.cgi?acc=GSE202124).

## Ethics Statement

The studies involving human participants were reviewed and approved by Ruijin Hospital, School of Medicine, Shanghai Jiao Tong University (2020104a). The patients/participants provided their written informed consent to participate in this study. The animal study was reviewed and approved by Shanghai Branch of Beijing Vital River Laboratory Animal Technologies Co. Ltd., China with authorization (P2022002).

## Author Contributions

ML and AZ contributed to the study concepts and study design. ML, XZ, and WZ contributed to the experimental studies and data acquisition. ML, MZ, and WL contributed to the literature survey. ML and WL contributed to the manuscript preparation, data analysis, and statistical analysis. AZ, BX, and QL contributed to the manuscript editing and review. All authors contributed to the article and approved the submitted version.

## Funding

This work was supported by the National Natural Science Foundation of China (No.81873857, No.82071596, No.82071712, and No.82101800), Shanghai Sailing Program (No.21YF1426600) and Shanghai Medical and Health Development Fund “Yiyuan Star Outstanding Young Physician” Project (SHWJRS(2021)-99).

## Conflict of Interest

The authors declare that the research was conducted in the absence of any commercial or financial relationships that could be construed as a potential conflict of interest.

## Publisher’s Note

All claims expressed in this article are solely those of the authors and do not necessarily represent those of their affiliated organizations, or those of the publisher, the editors and the reviewers. Any product that may be evaluated in this article, or claim that may be made by its manufacturer, is not guaranteed or endorsed by the publisher.

## Abbreviations

CEFFE, Cell-free fat extract; TEM, Transmission electron microscopy; DEGs, Differentially expressed genes; GO, Gene Ontology; KEGG, Kyoto Encyclopedia of Genes and Genomes; hGCs, human primary ovarian granulosa cells; HR, Homologous recombination; DDR, DNA damage repair.
