# Supplementary material for: Cell-Free Fat Extract Improves Ovarian Function and Fertility in Mice With Advanced Age
Source: Front Endocrinol (Lausanne). 2022 Jun 16;13:912648. doi: 10.3389/fendo.2022.912648 (PMC9243446; doi:10.3389/fendo.2022.912648)
Supplement: Supplementary file 1 [file DataSheet_1.docx]

Supplementary Material

# Supplementary Tables

Supplementary Table 1. Detailed information on the antibodies used

| **Antibody** | **Dilution ratio** | **Application** | **Product details** |
| --- | --- | --- | --- |
| P16 | 1:600 | IHC | ab241543, Abcam, UK |
| CD31 | 1:2000 | IHC | ab182981, Abcam, USA |
| Ki-67 | 1:200 | IHC | ab16667, Abcam, USA |
| PTEN | 1:200 | IHC | ab267787, Abcam, USA |
| P53 | 1:50 | IHC | ab131442, Abcam, USA |
| γH2AX | 1:200 | IHC | #9718, CST, USA |
| HRP-linked anti-rabbit IgG antibody | 1:100 | IHC | A0208, Beyotime, China |
| HRP-linked anti-mouse IgG antibody | 1:100 | IHC | A0216, Beyotime, China |
| GAPDH | 1:1000 | WB | #5174, CST, USA |
| P21 | 1:1000 | WB | #2947, CST, USA |
| P16 | 1:1000 | WB | Ab51243, Abcam, USA |
| γH2AX | 1:1000 | WB | #9718, CST, USA |
| HRP-linked anti-  rabbit IgG antibody | 1:3000 | WB | #7074, CST, USA |
| HRP-linked anti-mouse IgG antibody | 1:3000 | WB | #7076, CST, USA |
| FSHR | 1:50 | IF | 22665-1-AP, Proteintech, USA |
| Anti-Rabbit IgG - H&L (Alexa Fluor^@^488) | 1:500 | IF | #74412, CST, USA |
| Hoechst 33342 | 1:2000 | IF | C1022, Beyotime, China |

Supplementary Table 2. Primer sequences used for RT-PCR analysis

| **Gene**  **name** | **Species** | **Forward primer (5′ ➝ 3′)** | **Reverse primer (5′ ➝ 3′)** |
| --- | --- | --- | --- |
| *CCL2* | Human | CCTTCATTCCCCAAGGGCTC | GGTTTGCTTGTCCAGGTGGT |
| *CCL4* | Human | CTGTGCTGATCCCAGTGAATC | TCAGTTCAGTTCCAGGTCATACA |
| *SIRT2* | Human | GTCGCAGAGTCATCTGTTTGGT | AGTAGTGACAGATGGTTGGCTTGA |
| *IL1b* | Human | TTCTCTTCAGCCAATCTTCA | TGCCACTGTAATAAGCCATC |
| *P53* | Human | CGTGTGGAGTATTTGGATGAC | TGTGATGATGGTGAGGATGG |
| *GAPDH* | Human | GGAGTCCACTGGCGTCTTC | ATCTTGAGGCTGTTGTCATACTTC |

# Supplementary Figures


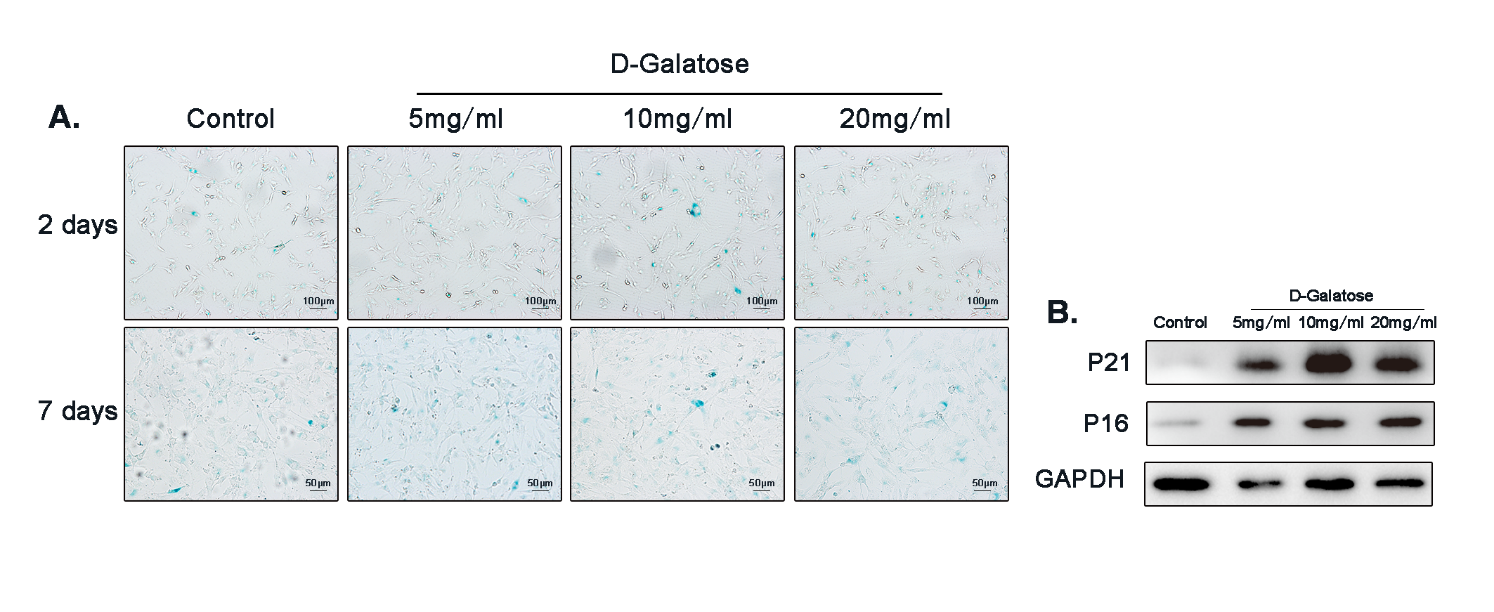


**Supplementary Figure 1. Construction the senescent KGN cells by D-gal. (A)** Construction of senescent KGN cells using 5,10, and 20 mg/mL D-gal for seven days and staining with β-Galactosidase. SA-β-gal-positive cells are shown in blue when observed under an optical microscope. **(B)** The expression of senescent protein marker (P21 and P16) was detected by western blotting.
